# Detection of Genital Pathogens and Co-Infections by Multiplex RT-qPCR: Associations with HIV Positivity and Demographic Factors

**DOI:** 10.3390/diagnostics16121793

**Published:** 2026-06-10

**Authors:** Murat Yaman, Gizem Nigdelioglu, Arzu Ilki

**Affiliations:** 1 Medical Microbiology, Marmara University Pendik Training and Research Hospital, Istanbul 34899, Türkiye; o.gzm4345@gmail.com (G.N.); ailki@marmara.edu.tr (A.I.); 2 Department of Medical Microbiology, Faculty of Medicine, Marmara University, Istanbul 34854, Türkiye

**Keywords:** genital infections, co-infections, HIV, multiplex RT-qPCR, sexually transmitted infections (STIs)

## Abstract

**Background/Objectives**: This study aimed to investigate the genital pathogen profile and co-infection dynamics using multiplex RT-qPCR, specifically evaluating the independent associations with age, sex, and HIV status. **Methods**: Data from 1217 patients who underwent a sexually transmitted infection (STI) panel study at the Microbiology Laboratory of Marmara University Pendik Training and Research Hospital between January 2024 and December 2025 were retrospectively reviewed. Pathogen detection was performed using a commercial kit (Bioeksen, Istanbul, Türkiye) with the multiplex RT-qPCR method. The independent effects of HIV positivity, age, and sex on pathogen frequency and co-infection were analyzed using logistic regression models; results were evaluated using adjusted odds ratio (aOR) and 95% confidence interval (CI). **Results**: Any-pathogen positivity was detected in 57.8% of patients, with *Ureaplasma* spp. (36.9%) and *Gardnerella vaginalis* (32.5%) being the most prevalent. While overall pathogen positivity did not differ significantly by HIV status (*p* = 0.158), HIV-positive patients exhibited distinct microbiological architectures. Higher positivity rates were observed in women in both groups, and a strong correlation was found between sex and the presence of infection (*p* < 0.001). Multivariate analysis revealed that HIV positivity was independently associated with an over fivefold increase in HSV-2 detection (aOR = 5.09, 95% CI: 1.47–17.65; *p* = 0.010). Furthermore, HIV-positive individuals were significantly enriched for complex polymicrobial patterns involving three or more pathogens (*p* = 0.008). Conversely, male sex was independently associated with a substantially lower risk of co-infection (aOR = 0.17, 95% CI: 0.12–0.22; *p* < 0.001), and increasing age showed an inverse relationship with co-infection frequency (aOR = 0.98 per year, 95% CI: 0.97–0.99; *p* = 0.001). **Conclusions**: This study adds to current epidemiological evidence by showing that genital pathogen distribution is shaped by age- and sex-related heterogeneity, with flora-associated co-infections predominating in women and more classical STI-related agents occurring more often in men. Our findings suggest that multiplex RT-qPCR provides value not only for broad pathogen detection but also for identifying demographic- and HIV-associated co-infection patterns that may support stratified screening and targeted clinical management.

## 1. Introduction

Genital infections remain a major challenge in both reproductive health and sexually transmitted infection (STI) control worldwide [1,2,3,4,5]. According to WHO estimates, curable STIs account for a substantial global burden, with nearly 1 million new infections occurring each day, while data from the USA suggest that approximately one in five adults experienced an STI in 2018 [1,6]. Global burden analyses further indicate that STI-related sequelae remain an important source of morbidity, underscoring the continuing public health relevance of these infections [6,7]. In response to this burden, international health authorities have emphasized comprehensive prevention and screening strategies aimed at interrupting transmission and improving early detection [2,4,8,9,10,11,12,13].

Many genital infections are asymptomatic or present with non-specific symptoms, which can delay diagnosis and treatment [14,15,16]. In addition, asymptomatic STIs and bacterial vaginosis have been shown to increase genital inflammation, disrupt mucosal barrier integrity, alter local cytokine responses, and facilitate the migration of pathogenic agents to the genital mucosa [8,9,10,11,16,17]. In this context, multiplex real-time polymerase chain reaction (RT-qPCR)-based diagnostic panels have improved the simultaneous detection of multiple genital pathogens from a single clinical sample [17,18,19,20]. These methods offer advantages not only for identifying classical STI agents but also for revealing co-infection patterns and polymicrobial profiles. Studies from different geographical settings have reported that rates of at least one detected pathogen range from 30% to 99%, whereas co-infection rates vary between 5% and 60% [21,22]. Multiplex RT-qPCR panel enables simultaneous detection of bacterial, viral, and protozoal genital pathogens from a single specimen, supporting rapid and broad molecular assessment. However, because RT-qPCR detects nucleic acids rather than viable organisms or clinical causality, positive results—especially for *Ureaplasma* spp. and Gardnerella vaginalis—should be interpreted within the patient’s symptoms, sample type, risk profile, and co-infection context.

The expansion of molecular diagnostic capacity has made it possible to examine the epidemiology of genital infections in greater detail. Previous studies suggest that pathogen distribution and co-infection patterns may vary according to age, sex, and HIV status [22,23,24]. However, most available reports focus on selected pathogens or restricted patient groups, and cohort-based analyses integrating demographic factors, HIV positivity, and polymicrobial detection within the same clinical population remain limited. Therefore, this study aimed to determine the distribution of pathogens detected by a multiplex genital infection panel, to examine variation according to demographic factors, and to evaluate the independent effect of HIV positivity on pathogen prevalence and co-infection.

## 2. Materials and Methods

### 2.1. Study Design and Population

This retrospective cross-sectional study was conducted using laboratory information management system (LIMS) data from all patients who underwent genital infection panel testing at the Microbiology Laboratory of Marmara University Pendik Training and Research Hospital between January 2024 and December 2025. A total of 1217 patients who underwent multiplex genital panel testing and had demographic data were included in the study.

Testing was performed as part of routine clinical practice in a symptomatic and/or clinician-requested population, rather than in a community-based screening setting. Repeat samples from the same patient were excluded from the analysis; each individual was evaluated only with the initial presentation sample.

All data were derived from routinely generated diagnostic results retrospectively retrieved from the LIMS and hospital records. No stored, preserved, or archived clinical specimens were reprocessed for this study. The laboratory workflow described below, therefore, reflects the routine diagnostic procedure applied at the time of clinical testing, not additional research-specific testing.

### 2.2. Laboratory Management

Urethral or vaginal swab specimens were submitted to the microbiology laboratory as part of routine clinical evaluation.

Nucleic acid extraction from urethral or vaginal swab samples was performed according to the manufacturer’s protocol using an automated extraction system (Zybio EXM 3000, Zybio Inc., Chongqing, China) and took approximately 10 min. The reaction mixture for amplification was prepared according to the Bio-Speedy^®^ Multiplex RT-qPCR panel (Bioeksen R&D Technologies, Istanbul, Türkiye) kit protocol [25]. Amplification was performed using a CFX96 Real-Time PCR System (Bio-Rad, Hercules, CA, USA) according to the manufacturer’s recommended thermal cycling protocol. The amplification protocol included the following thermal cycling conditions: Enzyme activation: 3 min at 52 °C (1 cycle); Pre-denaturation: 10 s at 95 °C (1 cycle). Following this, a touchdown PCR approach was applied to increase binding specificity: for 12 cycles: Denaturation: 1 s at 95 °C; binding/elongation: 15 s, starting at 67 °C and decreasing by 1 °C in each cycle to 56 °C. Amplification after the touchdown phase: for 30 cycles: Denaturation: 1 s at 95 °C and binding/elongation: 15 s at 55 °C. During amplification, fluorescent signals were measured simultaneously on the appropriate channels of the instrument (FAM, HEX, ROX, and Cy5). Results were analyzed using Bio-Rad CFX Maestro Software version 2.3, and evaluation was performed according to the threshold and cut-off values recommended by the manufacturer. In positivity assessment, the amplification curve and Ct (cycle threshold) values were considered together. Genital pathogens qualitatively sought with this method are shown in Table 1. Ureaplasma species were grouped under a single variable (*Ureaplasma* spp.) during the analysis. Detection of two or more pathogens in the same patient was defined as “co-infection”.

### 2.3. Variable Definitions

Demographic variables included age, sex, and HIV status. HIV status was defined according to the final confirmed result recorded in the hospital information system, with confirmation performed through the national diagnostic algorithm. Age was analysed as a continuous variable and, when required, categorized into pediatric/adolescent (<18 years) and adult (≥18 years) groups.

Pathogen positivity was defined as qualitative detection of pathogen-specific nucleic acid by multiplex RT-qPCR. The pathogen profile referred to the sample-level pattern of detected pathogens, including single and multiple detections. Single-pathogen detection was defined as positivity for one pathogen only. Molecular co-infection was defined as the detection of two or more pathogens in the same specimen; double co-infection indicated exactly two pathogens, and complex co-infection indicated three or more pathogens. Because RT-qPCR detects nucleic acid rather than pathogen viability or clinical causality, flora-associated organisms such as *Ureaplasma* spp. and *Gardnerella vaginalis* were interpreted as molecular detections requiring clinical correlation. The term “co-infection” was retained in selected tables and headings to remain consistent with common clinical terminology.

### 2.4. Statistical Analysis

Statistical analyses were performed using IBM SPSS Statistics software (version 27; IBM Corp., Armonk, NY, USA) and Python (v3.11) after data cleaning and configuration, using the pandas and statsmodels libraries. Descriptive statistics are presented as mean ± standard deviation or number (percentage). The chi-square test or Fisher’s exact test, as appropriate, was used for categorical comparisons; exact tests were applied when expected cell counts were small. The Mann–Whitney U test was used to compare age distribution between genders. To assess independent associations, separate multivariable logistic regression models were constructed for overall co-infection and for selected pathogen-specific outcomes, with HIV status, sex, and age entered simultaneously as covariates. Results are reported as adjusted odds ratios (aORs) with 95% confidence intervals (CIs). A two-sided *p*-value of <0.05 was considered statistically significant.

As this study was based on molecular detection, the presence of some flora-associated organisms—particularly *Ureaplasma* spp. and Gardnerella vaginalis—should be interpreted with caution, as molecular positivity may represent colonization as well as clinically overt infection.

### 2.5. Ethical Approval

This retrospective cross-sectional study was approved by the Marmara University Faculty of Medicine Clinical Research Ethics Committee (approval date/number: 25.09.2025/25-338) and conducted in accordance with the principles of the Declaration of Helsinki. All data used in the study were analyzed in an anonymized form, and the identities of the participants were kept confidential.

## 3. Results

### 3.1. Demographic Characteristics

A total of 1217 patients were included in the study. Of these, 725 (59.6%) were male, and 492 (40.4%) were female, with a mean age of 32.42 ± 13.11 years. HIV positivity was identified in 109 patients (9.0%), and 105 of these cases (96.3%) were in the adult age group. Compared with HIV-negative patients, HIV-positive patients were significantly more likely to be male (84.4% vs. 57.1%, *p* < 0.001) and were older on average (35.87 ± 11.84 vs. 32.08 ± 13.19 years, *p* = 0.002). The comparative demographic characteristics of the HIV-positive and HIV-negative groups are presented in Table 2.

### 3.2. Pathogen Distribution and Its Relationship with HIV Status

Any-pathogen positivity was detected in 703 of 1217 patients (57.8%). Single-pathogen detection was identified in 413 patients (33.9%), whereas co-infection, defined as the detection of two or more pathogens, was identified in 290 patients (23.8%). Among HIV-positive patients, single-pathogen detection was observed in 27 of 109 cases (24.8%), compared with 386 of 1108 HIV-negative cases (34.8%). Although HIV status was not significantly associated with single-pathogen detection, the overall distribution pattern differed across HIV strata, with HIV-positive patients showing a lower proportion of single detections and a higher proportion of triple-or-more detections.

Across both HIV strata, pathogen positivity was consistently more frequent in women than in men. In the HIV-negative group, any-pathogen positivity was detected in 118 of 143 females aged <18 years (82.6%) and in 262 of 332 adult females (78.9%), compared with 3 of 6 males aged <18 years (50.0%) and 264 of 627 adult males (42.1%). A similar pattern was observed in the HIV-positive group, in which any-pathogen positivity was detected in 13 of 14 adult women (92.9%) and 40 of 91 adult men (44.0%). These findings indicate marked sex-related heterogeneity in pathogen burden, with the highest positivity rates observed in women, particularly adult women. The detailed distribution of single, double, and triple-or-more detections according to HIV status, sex, and age is shown in Table 3. Small subgroup counts in younger HIV-positive patients should be interpreted descriptively.

### 3.3. Co-Infection Dynamics

Co-infection, defined as the detection of two or more pathogens, was identified in 290 of 1217 patients (23.8%). The crude co-infection rate was 26.6% in the HIV-positive group and 23.6% in the HIV-negative group. Among HIV-positive adults, co-infection was markedly more frequent in women than in men, affecting 9 of 14 women (64.3%) and 18 of 91 men (19.8%), further supporting a pronounced sex-based gradient in co-infection burden. In univariable comparisons, co-infection was more frequent in women than in men (*p* < 0.01). In multivariable analysis, male sex was independently associated with a lower likelihood of co-infection (aOR = 0.17; 95% CI: 0.12–0.22; *p* < 0.001), whereas increasing age was inversely associated with co-infection frequency (aOR = 0.98; 95% CI: 0.97–0.99; *p* = 0.001). By contrast, HIV positivity was not identified as an independent predictor of overall co-infection (aOR = 0.88; 95% CI: 0.51–1.53; *p* = 0.65). These findings indicate that overall co-infection burden in this cohort was more strongly associated with demographic factors, particularly sex and age, than with HIV status.

### 3.4. Overall Pathogen Spectrum

**Figure 1:**
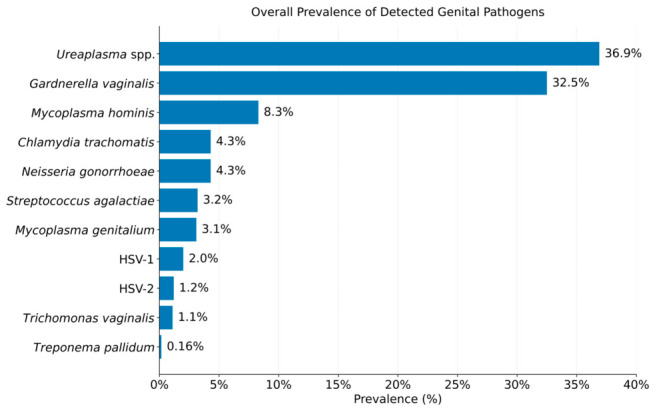
Overall Prevalence of Detected Genital Pathogens. Note: Data are presented as percentages of the total cohort (*n* = 1217).

The prevalence of the most frequently detected pathogens is shown in Figure 1. The two most prevalent organisms in the overall cohort were *Ureaplasma* spp. (36.9%) and *Gardnerella vaginalis* (32.5%). This distribution indicates that the pathogen spectrum in this cohort was dominated by flora-associated organisms rather than by less frequently detected classical STI pathogens. Data in Figure 1 are presented as percentages of the total cohort (*n* = 1217).

### 3.5. Pathogen Distribution According to HIV Status

To provide a focused comparison across HIV strata, Table 4 presents three representative pathogens selected on the basis of their relevance to the study findings: *Ureaplasma* spp. as the most prevalent flora-associated organism, Gardnerella vaginalis as a second major flora-associated comparator, and HSV-2 as the pathogen showing the most distinct association with HIV positivity. The overall prevalence of HSV-2 was found to be 1.15% (14/1217). When stratified by HIV status, HSV-2 was detected in 4 of 109 HIV-positive patients (3.7%) and in 10 of 1108 HIV-negative patients (0.9%) (Table 4). In contrast, *Ureaplasma* spp. was detected less frequently in HIV-positive than in HIV-negative patients (21/109 [19.3%] vs. 428/1108 [38.6%], *p* = 0.037), whereas *G. vaginalis* showed a comparable prevalence in the two groups (33/109 [30.3%] vs. 363/1108 [32.8%], *p* = 0.593). Thus, among the selected pathogens evaluated individually, HSV-2 showed the clearest enrichment in the HIV-positive group, whereas *Ureaplasma* spp. displayed an inverse pattern, and *G. vaginalis* remained relatively stable across HIV strata.

In multivariable logistic regression analysis adjusted for age and sex, HIV positivity remained independently associated with HSV-2 detection (aOR = 5.09; 95% CI: 1.47–17.65; *p* = 0.010). By contrast, an inverse independent association was observed between *Ureaplasma* spp. detection and HIV positivity (aOR = 0.58; 95% CI: 0.35–0.97; *p* = 0.037). No significant independent association was found between HIV positivity and the other evaluated pathogens, including *G. vaginalis* (*p* > 0.05). These findings identify HSV-2 as the most distinctive HIV-associated pathogen signal in the cohort.

**Figure 2:**
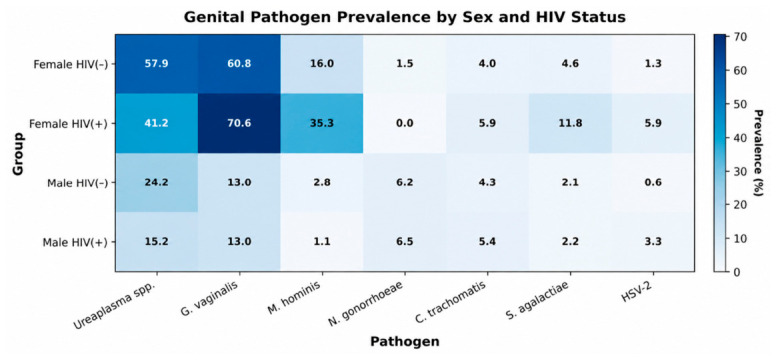
Heat map representation of genital pathogen prevalence according to sex and HIV status. Color intensity represents pathogen prevalence; low prevalence is shown with white/gray tones, and high prevalence with blue and midnight blue tones. Since more than one pathogen can be detected in the same sample, the sum of the percentages can exceed 100%.

When examined across age and sex subgroups, *G. vaginalis* and *Ureaplasma* spp. were particularly frequent in women. In men, especially in the adult age group (≥18 years), relatively higher proportions of *N. gonorrhoeae*, *C. trachomatis*, and HSV-2 were observed in HIV-positive individuals; however, among these pathogens, only HSV-2 showed a statistically significant association with HIV status. The subgroup distribution of pathogen frequencies is illustrated in Figure 2.

### 3.6. Polymicrobial Combination Patterns

The distribution of polymicrobial combinations according to HIV status is presented in Table 5. Among patients with co-infection, the most frequent double combination in both HIV strata was *Ureaplasma* spp. + *G. vaginalis*, accounting for 98 of 261 polymicrobial infections (37.5%) in the HIV-negative group and 6 of 29 (20.7%) in the HIV-positive group. Although this difference did not reach statistical significance in univariable analysis (*p* = 0.324), multivariable analysis adjusted for age and sex showed that the co-occurrence of *Ureaplasma* spp. + *G. vaginalis* was independently inversely associated with HIV positivity (aOR = 0.31; 95% CI: 0.16–0.61; *p* < 0.001).

Among polymicrobial infections, the most frequent double combination in both HIV strata was *Ureaplasma* spp. + *G. vaginalis*. Although this pattern was numerically more common in HIV-negative than in HIV-positive patients (37.5% vs. 20.7%), the difference was not significant in univariable analysis (*p* = 0.324). However, in multivariable analysis adjusted for age and sex, this co-occurrence showed an independent inverse association with HIV positivity (aOR = 0.31; 95% CI: 0.16–0.61; *p* < 0.001). In contrast, HIV-positive patients exhibited more complex polymicrobial patterns, including triple-or-more combinations (37.9% vs. 23.8%; *p* = 0.008) and HSV-2-containing profiles, particularly *Ureaplasma* spp. + *G. vaginalis* + HSV-2 (13.8% vs. 4.6%; *p* = 0.002).

## 4. Discussion

This study provides a stratified analysis of genital pathogen detection in a large hospital-based cohort evaluated by multiplex RT-qPCR, showing that pathogen burden was influenced not only by HIV status but also, and in several analyses more strongly, by sex and age. The overall pathogen spectrum was dominated by flora-associated organisms, particularly *Ureaplasma* spp. and *Gardnerella vaginalis*, whereas HSV-2 emerged as the most distinctive HIV-associated pathogen signal. Women carried a substantially higher burden of pathogen positivity and co-infection than men, while HIV-positive patients showed a relative enrichment of more complex and HSV-2-containing polymicrobial profiles. Together, these findings suggest that genital infections in this cohort are better understood as recurring microbiological patterns rather than isolated pathogen-specific events [8,9,15,18].

The predominance of *Ureaplasma* spp. and *G. vaginalis* is consistent with previous studies showing that flora-associated organisms account for a major proportion of genital pathogen detections in both symptomatic and mixed clinical populations [19,24,26,27,28]. Similar findings have been reported in multiplex PCR-based studies from Europe as well as in symptomatic male populations from Türkiye [26,27,28,29]. In our study, the higher detection rates of these organisms in women are biologically plausible and may reflect the vaginal microenvironment, where dysbiosis, reduced lactobacillus dominance, and mucosal inflammation can facilitate microbial persistence and co-infection [7,8,12,13,30]. Data from Italy and South Korea are also consistent with the observation that co-infections in women frequently involve these flora-associated agents [28,30,31].

By contrast, *Mycoplasma genitalium*, *N. gonorrhoeae*, and *C. trachomatis* were relatively more frequent in male subgroups, particularly among HIV-positive adults. Similar male-skewed detection patterns have been reported in studies from New Zealand, Israel, and Türkiye [32,33,34,35]. However, these findings should be interpreted cautiously. Higher detection in men may reflect not only biological or behavioral differences, but also differential symptom burden and test-seeking patterns, especially in urethritis-oriented clinical settings. Accordingly, our data are best understood as reflecting the epidemiology of a tested hospital population rather than direct community prevalence.

The strongest pathogen-specific association in this study was the link between HIV positivity and HSV-2. HSV-2 was detected approximately fourfold more frequently in HIV-positive than in HIV-negative patients, and this relationship remained significant after adjustment for age and sex. A similarly strong association has been reported in a study from the USA, further supporting the robustness of the HIV–HSV-2 link observed in our cohort [36]. This finding is biologically plausible and consistent with the extensive literature describing bidirectional epidemiological synergy between HIV and HSV-2, mediated through mucosal barrier disruption, inflammatory recruitment, and enhanced viral susceptibility and shedding [11,36,37,38,39,40,41]. In our dataset, HSV-2 behaved differently from flora-dominant organisms: it was selectively enriched in the HIV-positive subgroup rather than merely common. This makes HSV-2 the clearest HIV-associated pathogen signal identified in the present study [36,37,38,39,40,41].

An equally important finding was that HIV positivity did not independently predict overall co-infection burden. Instead, co-infection was more strongly associated with female sex and younger age. This argues against a simplistic interpretation in which HIV alone explains polymicrobial genital infection. Rather, the probability of co-infection in this cohort appears to be shaped primarily by demographic factors and the broader genital microbial context [42,43,44,45]. At the same time, HIV status remained relevant to the structure of polymicrobial detection. Although HIV positivity did not independently increase overall co-infection, it was associated with a shift toward more complex polymicrobial architectures, including triple-or-more combinations and HSV-2-containing profiles. In other words, HIV in this cohort appears to modify co-infection structure more than co-infection frequency itself.

This distinction is particularly evident in the polymicrobial analyses. The most frequent double combination in both HIV strata was *Ureaplasma* spp. plus *G. vaginalis*, yet this pattern showed an inverse adjusted association with HIV positivity. By contrast, HSV-2-containing combinations—especially *Ureaplasma* spp. + *G. vaginalis* + HSV-2—were more prevalent in HIV-positive patients. These findings suggest that some co-infection patterns may reflect background genital microbial ecology, whereas others may be more closely linked to HIV-associated microbiological clustering. From a diagnostic standpoint, this is where multiplex RT-qPCR may add particular value, by capturing not only which pathogens are present but also how they cluster within potentially clinically meaningful patterns [19,20,21,22,23,24,25,26].

These observations may also be relevant for how multiplex panel results are interpreted in routine clinical practice. In populations undergoing genital pathogen testing, particularly where HIV burden is non-negligible, interpretation should move beyond single-agent positivity. Sex, age, HIV status, and polymicrobial structure should be considered together. The enrichment of HSV-2 and HSV-2-containing polymicrobial profiles in HIV-positive patients may support integrated STI assessment within HIV-focused care pathways, while the strong demographic patterning observed in this cohort highlights the need for stratified interpretation of multiplex panel results [7,13,22,26]. Clinically, our findings support the interpretation of multiplex RT-qPCR results within a syndromic, risk-based, and patient-contextual framework rather than as isolated laboratory endpoints. Isolated detection of *Ureaplasma* spp. or *Gardnerella vaginalis* should not automatically be regarded as an indication for antimicrobial treatment, because these organisms may represent colonization or genital dysbiosis as well as clinically relevant infection. Their clinical significance is likely to increase in symptomatic patients, recurrent presentations, pregnancy-related contexts, or when classical STI pathogens are co-detected. Conversely, HSV-2 positivity in HIV-positive individuals may identify a subgroup requiring more integrated STI–HIV assessment, particularly when accompanied by genital symptoms, recurrent lesions, additional STI co-infections, or complex polymicrobial profiles. In such cases, HSV-2 detection should prompt broader STI evaluation, sexual risk counselling, partner management when appropriate, and reinforcement of linkage to optimized HIV care.

This study has limitations. Its retrospective, single-center design limits generalizability, and the tested cohort may not reflect community-level epidemiology. Because testing was performed in a symptomatic and/or clinician-requested population rather than in a community screening cohort, pathogen frequencies and co-infection patterns may have been influenced by referral practices, symptom-driven testing, clinician suspicion, HIV-risk profile, sex distribution, and healthcare-seeking behaviour. Therefore, the results should not be interpreted as community-level prevalence estimates, but rather as findings applicable to clinically selected patients undergoing genital pathogen testing in a tertiary-care setting. Clinical symptom data, behavioural variables, and HIV-specific markers such as antiretroviral treatment status, CD4 count, and viral load were not available. In addition, molecular detection of flora-associated organisms such as *Ureaplasma* spp. and *G. vaginalis* does not necessarily distinguish colonization from clinically overt infection. Nevertheless, the study also has important strengths, including a relatively large real-world cohort, use of a uniform multiplex molecular platform, and the application of stratified and multivariable analyses that enabled the identification of pathogen-specific and polymicrobial patterns beyond simple prevalence estimates [19,20,21,22,23,24,25,26,44,45,46].

In conclusion, genital pathogen detection in this cohort was characterized by a predominance of flora-associated organisms, marked sex-related heterogeneity, and distinct HIV-associated patterns, particularly for HSV-2. Beyond the simultaneous detection of multiple STI pathogens, multiplex RT-qPCR, when interpreted alongside HIV status and demographic characteristics, may offer meaningful insight into the epidemiological patterning of genital infections and support a more nuanced and clinically informative diagnostic assessment.

## Figures and Tables

**Table 1:** Classification of pathogens included in the genital multiplex RT-qPCR panel.

Type of Pathogen	Pathogens
Bacterial	*Streptococcus agalactiae*, *Gardnerella vaginalis*, *Neisseria gonorrhoeae*, *Treponema pallidum*, *Ureaplasma parvum*/*Ureaplasma urealyticum*, *Mycoplasma genitalium*, *Mycoplasma hominis*, *Chlamydia trachomatis*
Viral	*Herpes Simplex Virus 1* (*HSV-1*), *Herpes Simplex Virus 2* (*HSV-2*)
Protozoal	*Trichomonas vaginalis*

**Table 2:** Comparison of demographic characteristics between HIV-positive and HIV-negative patients.

Demographic Characteristics	HIV-Positive (*n* = 109)	HIV-Negative (*n* = 1108)	*p*-Value
Male, *n* (%)	92 (84.4)	633 (57.1)	<0.001
Female, *n* (%)	17 (15.6)	475 (42.9)	<0.001
Age, mean ± SD, y	35.87 ± 11.84	32.08 ± 13.19	0.002

Data are presented as *n* (%) or mean ± SD. SD, standard deviation. *n*, number of cases; y, years.

**Table 3:** Pattern of single and multiple pathogen detections stratified by HIV status, sex, and age.

Group	Total, *n*	Single, *n* (%)	Double, *n* (%)	≥3, *n* (%)	Any Positive, *n* (%)
HIV-negative	1108	386 (34.8)	199 (18.0)	62 (5.6)	647 (58.4)
Female, <18 y	143	58 (40.6)	42 (29.4)	18 (12.6)	118 (82.6)
Female, ≥18 y	332	142 (42.8)	88 (26.5)	32 (9.6)	262 (78.9)
Male, <18 y	6	2 (33.3)	1 (16.7)	0 (0.0)	3 (50.0)
Male, ≥18 y	627	184 (29.3)	68 (10.8)	12 (1.9)	264 (42.1)
HIV-positive	109	27 (24.8)	18 (16.5)	11 (10.1)	56 (51.4)
Female, <18 y	3	1 (33.3)	1 (33.3)	1 (33.3)	3 (100.0)
Female, ≥18 y	14	4 (28.6)	5 (35.7)	4 (28.6)	13 (92.9)
Male, <18 y	1	0 (0.0)	0 (0.0)	0 (0.0)	0 (0.0)
Male, ≥18 y	91	22 (24.2)	12 (13.2)	6 (6.6)	40 (44.0)

Any positive indicates the number and proportion of individuals with at least one detected pathogen. *n*, number of cases; y, years.

**Table 4:** Comparison of selected pathogen frequencies between HIV-positive and HIV-negative patients.

Pathogen	HIV-Positive, *n* (%)	HIV-Negative, *n* (%)	*p*-Value	Statistical Test
*Ureaplasma* spp.	21 (19.3)	428 (38.6)	0.037	Chi-square
*Gardnerella vaginalis*	33 (30.3)	363 (32.8)	0.593	Chi-square
*HSV-2*	4 (3.7)	10 (0.9)	0.030	Fisher’s exact test

**Table 5:** Distribution of double and triple-or-more pathogen combinations among patients with polymicrobial infection, stratified by HIV status.

Combination Pattern	HIV-Negative (*n* = 261), *n* (%)	HIV-Positive (*n* = 29), *n* (%)	*p*-Value
Double combinations, overall	199 (76.2)	18 (62.1)	0.142
*Ureaplasma* spp. + *G. vaginalis*	98 (37.5)	6 (20.7)	0.324
*Ureaplasma* spp. + *M. hominis*	42 (16.1)	4 (13.8)	0.650
*G. vaginalis* + *M. hominis*	35 (13.4)	3 (10.3)	0.782
Other double combinations	24 (9.2)	5 (17.2)	0.045
Triple-or-more combinations, overall	62 (23.8)	11 (37.9)	0.008
*Ureaplasma* spp. + *G. vaginalis* + *M. hominis*	38 (14.6)	5 (17.2)	0.115
*Ureaplasma* spp. + *G. vaginalis* + HSV-2	12 (4.6)	4 (13.8)	0.002
Other polymicrobial combinations	12 (4.6)	2 (6.9)	0.450

Note: Patient numbers are presented as *n*. Data are presented as *n* (%). Percentages were calculated using the number of patients with polymicrobial infection in each HIV stratum as denominators (HIV-negative, *n* = 261; HIV-positive, *n* = 29). Comparisons were performed using the chi-square test or Fisher’s exact test, as appropriate. Percentages may not sum exactly to overall values because of rounding to one decimal place.

## Data Availability

The original contributions presented in this study are included in the article. Further inquiries can be directed to the corresponding author.
